# Headaches

**Published:** 1871-10

**Authors:** 


Headaches: Their Causes and their Cure. By Henry G. Wright, M. D., etc. From the fourth London edition. Lindsay & Blakiston, Philadelphia.

This little work, which has been for some time out of print, is already well known to the profession, and requires nothing more than the mention of its re-issue to insure to it a favorable reception.

